# Modelling spatial trends in sorghum breeding field trials using a two-dimensional P-spline mixed model

**DOI:** 10.1007/s00122-017-2894-4

**Published:** 2017-04-03

**Authors:** Julio G. Velazco, María Xosé Rodríguez-Álvarez, Martin P. Boer, David R. Jordan, Paul H. C. Eilers, Marcos Malosetti, Fred A. van Eeuwijk

**Affiliations:** 1 Biometris, Wageningen University and Research, P.O. Box 16, 6700 AA Wageningen, The Netherlands; 2 Department of Plant Breeding, National Institute of Agricultural Technology (INTA), B2700WAA EEA Pergamino, Buenos Aires, Argentina; 3 BCAM, Basque Center for Applied Mathematics, Bilbao, Spain; 4 IKERBASQUE, Basque Foundation for Science, Bilbao, Spain; 5 Queensland Alliance for Agriculture and Food Innovation, Hermitage Research Facility, The University of Queensland, Warwick, QLD 4370 Australia; 6 Erasmus University Medical Centre, Rotterdam, The Netherlands

## Abstract

****Key message**:**

***A flexible and user-friendly spatial method called SpATS performed comparably to more elaborate and trial-specific spatial models in a series of sorghum breeding trials***.

**Abstract:**

Adjustment for spatial trends in plant breeding field trials is essential for efficient evaluation and selection of genotypes. Current mixed model methods of spatial analysis are based on a multi-step modelling process where global and local trends are fitted after trying several candidate spatial models. This paper reports the application of a novel spatial method that accounts for all types of continuous field variation in a single modelling step by fitting a smooth surface. The method uses two-dimensional P-splines with anisotropic smoothing formulated in the mixed model framework, referred to as SpATS model. We applied this methodology to a series of large and partially replicated sorghum breeding trials. The new model was assessed in comparison with the more elaborate standard spatial models that use autoregressive correlation of residuals. The improvements in precision and the predictions of genotypic values produced by the SpATS model were equivalent to those obtained using the best fitting standard spatial models for each trial. One advantage of the approach with SpATS is that all patterns of spatial trend and genetic effects were modelled simultaneously by fitting a single model. Furthermore, we used a flexible model to adequately adjust for field trends. This strategy reduces potential parameter identification problems and simplifies the model selection process. Therefore, the new method should be considered as an efficient and easy-to-use alternative for routine analyses of plant breeding trials.

## Introduction

Efficient phenotypic and genomic selection schemes in plant breeding programs rely on accurate assessment of the phenotypic performance of genotypes in field experiments (Qiao et al. 2004; Lado et al. 2013; Bernal-Vasquez et al. 2014; Sarker and Singh 2015). Plant breeding trials usually involve a large number of test entries covering large areas where spatial variation is likely to be an obstacle to reliable prediction of genetic values. This is particularly challenging in early generation variety trials conditioned by the use of limited replication of genetic material.

A number of sophisticated experimental designs, such as those enabling the recovery of inter-block information (Yates 1940; Patterson et al. 1978; John and Williams 1995) or partially replicated designs (Cullis et al. 2006; Williams et al. 2014), have been developed to correct for part of the field trend. However, efficient approaches to account for more complex environmental variation require complementing experimental designs with appropriate models of analysis (Basford et al. 1996; Qiao et al. 2000; Smith et al. 2002). Several spatial methods have been suggested to improve the precision of phenotyping. The most commonly used spatial models consider the correlation between residuals from neighboring plots to adjust for local trend or small-scale variation. These spatial methods include nearest neighbor analyses (Bartlett 1978; Wilkinson et al. 1983), and mixed model analyses using the first-order autoregressive (AR1) functions (Cullis and Gleeson 1991) or other spatial covariance structures (e.g. Zimmerman and Harville 1991; Piepho and Williams 2010). Polynomials have been also used on top of experimental design features to account for additive and non-additive trends along row and column directions (Edmondson 1993; Federer 1998). Fertility trends in early generation variety trials have been modelled by fitting one-dimensional cubic smoothing splines within blocks (Durbán et al. 2001). Durbán et al. (2003) applied semiparametric models for spatial analysis of field experiments and presented graphical and analytical model selection criteria.

Within the mixed model framework, Gilmour et al. (1997) proposed an elaborate procedure for spatial analysis of agricultural variety trials. Their approach starts by fitting a two-dimensional separable AR1 model by default to account for local trend. Eventually, extraneous variation resulting from trial management practices may be accommodated with additional model terms, while global trends reflecting large-scale variation across the field are modelled by one-dimensional polynomials or splines in the direction of rows and/or columns. The authors suggested a sequential model-fitting scheme to identify the most suitable spatial model. The procedure relies on graphical diagnostic tools and requires several modelling choices to be tried. Stefanova et al. (2009) extended this modelling process by including more formal diagnostics to facilitate model selection. However, the above-mentioned approach is not without limitations. First and foremost, the proposed multi-step procedures may not be attractive for routine analysis of large series of trials, since it requires a high level of hands-on intervention. Furthermore, there exists a risk of over-fitting the spatial data when the number of candidate models involved in the model selection process increases. Finally, convergence failures due to parameter identification problems may occur when trying to fit different spatial terms simultaneously (Dutkowski et al. 2006; Müller et al. 2010; Piepho et al. 2015).

Multidimensional regression spline methods represent a flexible alternative to account for complex variation structures. They allow the modelling of smooth multidimensional (or interaction) surfaces (e.g., Ruppert et al. 2003; Currie et al. 2006; Wood 2006). Regression splines are efficient curve-fitting functions composed of polynomial pieces, generally quadratic or cubic, that are joined at points called “knots”. An interesting method using splines is based on two-dimensional P-splines (2D P-splines) as proposed by Eilers and Marx (1996, 2003), and its formulation in the linear mixed model framework (Eilers 1999; Currie and Durbán 2002). P-splines combine regression splines and a roughness penalty, which is the key component. This penalization is tuned by one or more smoothing parameters that control the degree of smoothness of the fitted spatial surface to prevent over-fitting. The connection between P-splines and mixed models provides attractive advantages. It enables the use of efficient algorithms for inference and prediction. Furthermore, the optimal smoothing parameters are automatically estimated by restricted maximum likelihood (REML; Patterson and Thompson 1971) as ratios of variance components.

Some applications of 2D P-spline models have been reported for spatial analysis of field trials. Cappa and Cantet (2007) and Cappa et al. (2011, 2015) used these models within a Bayesian approach to account for global trends in forest genetic trials. These studies considered a single smoothing parameter that controls the smoothness of the spatial effects in the direction of both rows and columns, imposing isotropic smoothing. In agricultural experiments, Taye and Njuho (2008) proposed using P-splines in two dimensions to adjust for global trend and to model local variation with Papadakis and kriged covariates. The authors compared P-spline models assuming additive trends or interaction between trends and emphasized the importance of choosing between both model settings. A different approach to spatial analysis of field trials using 2D P-spline mixed models was recently proposed by Rodríguez-Álvarez et al. (2016a). They introduced a novel spatial model that adjusts for both global and local trends simultaneously. The authors called this model SpATS, an acronym for Spatial Analysis of field Trials with Splines. The new spatial method makes use of the P-spline ANOVA representation of the smooth surface according to Lee et al. (2013). The distinctive feature of the SpATS model is an attractive decomposition of the spatial surface into additive one-dimensional trends and two-dimensional interaction trends. Furthermore, the model assigns a different smoothing parameter to each spatial component, allowing for anisotropic smoothing. This parametrization enables a flexible modelling of the spatial surface, where each component has a straightforward interpretation.

For the present research, we considered a series of multi-environmental trials from a sorghum [*Sorghum bicolor* (L.) Moench] breeding program in eastern Australia. These trials belong to the initial stages of evaluations, where a large number of breeding lines (approximately 1000) were tested in each experiment using partially replicated designs. Furthermore, studies regarding the implications of performing spatial analysis in sorghum genetic trials are limited in the literature. Consequently, this data set serves to illustrate a situation when a flexible and efficient spatial analysis tool is specially required.

This paper reports an application of the SpATS mixed model to adjust for all types of field trend in early generation sorghum breeding trials. We use a one-step modelling approach to spatial analysis by fitting a general SpATS model to analyze the whole series of trials. This approach is assessed in comparison with more elaborate and trial-specific spatial models identified according to the method of Gilmour et al. (1997). Both methods are compared in terms of variance component estimates, the improvement of precision, and correlation of predicted genotypic effects. The new spatial model has been fitted using a tailor-made R package (R Development Core Team 2016) called SpATS (Rodríguez-Álvarez et al. 2016b), which is publicly available from CRAN (https://cran.r-project.org/package=SpATS).

## Materials and methods

### Data set

In this study, we used data from 21 sorghum breeding trials conducted at 12 different locations in eastern Australia between 2005 and 2008. The data set is part of the public germplasm enhancement program managed by the University of Queensland and Queensland’s Department of Agriculture and Fisheries. A total of 3947 backcross recombinant inbred lines (BC-RILs) were evaluated as male parents in test-cross hybrid combinations with a single female tester. The BC-RILs were derived from crosses between an elite inbred line and a range of exotic sorghum lines. Detailed descriptions of the breeding population used in this paper can be found in Jordan et al. (2011) and Mace et al. (2013). The set of trials is considered to represent the target population of environments in the Australian sorghum cropping region.

Each trial was laid out as a rectangular array using resolvable *p*-rep designs (Cullis et al. 2006). Table 1 summarizes information related to the individual trials, including the field layout and the number of genotypes per location. Plots were 5 m wide along rows by 1.5 or 2 m long down the columns, with two rows of plants in each plot. The *p*-rep designs consisted of 30% of the test-cross hybrids having two replicates (*p* = 30%), while the remaining 70% of the genotypes were unreplicated. Across all trials, a total of ten commercial varieties were included as check entries with additional levels of replication. Allocation of the replicated test genotypes was based on an optimality measure determined by the average pairwise prediction error variance and assuming a pre-specified spatial model (Cullis et al. 2006). The search algorithm is constrained, so that the replicated hybrids occurred once in each half of the trial, which established two resolvable blocks in all the designs.

**Table 1:** Description of experimental layout and mean values of grain yield (GY) and plant height (PH) for each trial in the sorghum breeding data set

Trial	Year	Location	Rows	Columns	Plots	Genotypes	Mean GY (t/ha)	Mean PH (cm)
BIL05	2005	Biloela	55	28	1540	1136	4.10	104
DAB05	2005	Dalby Box	76	20	1520	1167	3.05	91
DYS05	2005	Dysart	77	20	1540	1079	1.31	95
HER05	2005	Hermitage	77	20	1540	1202	7.65	113
JIM05	2005	Jimbour	44	20	880	682	4.60	105
BIL06	2006	Biloela	81	20	1620	1060	6.70	115
CEP06	2006	Cecil Plains	48	30	1440	953	3.24	95
DAB06	2006	Dalby	62	20	1240	823	2.00	101
GON06	2006	Goondiwindi	72	20	1440	957	6.58	117
HER06	2006	Hermitage	74	20	1480	1075	8.75	109
BIL07	2007	Biloela	86	20	1720	998	2.78	104
CLE07	2007	Clermont	34	40	1360	768	3.03	112
DYS07	2007	Dysart	44	40	1760	938	2.82	113
HER07	2007	Hermitage	70	25	1750	1012	5.39	104
BIL08	2008	Biloela	80	20	1600	1010	4.33	123
DAB08	2008	Dalby Box	64	20	1280	947	6.67	133
DAL08	2008	Dalby	62	20	1240	903	6.48	132
HER08	2008	Hermitage	80	20	1600	1012	–	133
KIL08	2008	Kilcummin	75	20	1500	899	3.36	131
LIV08	2008	Liverpool Plains	66	20	1320	980	10.06	125
SPR08	2008	Springsure	46	20	920	753	3.87	–

We illustrate the spatial analyses with two traits: grain yield (t/ha) and plant height (cm). Data were not available for grain yield at trial HER08 and for plant height at trial SPR08 (Table 1). The proportion of missing plots ranged between 3 and 29%.

### The SpATS model

In this section, we present a brief description of the SpATS model; for a thorough treatment of the model specifications, we refer to the original study by Rodríguez-Álvarez et al. (2016a).

Consider that observations in each sorghum breeding trial were obtained from plots arranged as a rectangular grid, where plot positions are collected in vectors of row (***r***) and column (***c***) coordinates. Under the SpATS model, field trends are modelled by a smooth bivariate function of the spatial coordinates $f({\varvec{r}},~{\varvec{c}})$ represented by 2D P-splines. As said, this technique optimizes the fitted surface by penalizing or shrinking the spatial effects. The magnitude of the penalization over the fitted trend is determined by the smoothing parameters. These terms control the balance between smoothness of the fitted surface and fidelity to the spatial data. For instance, larger values of the smoothing parameters result in smoother spatial gradients, while smaller values produce rougher fitted trends. Following the approach of Rodríguez-Álvarez et al. (2016a), additional terms were included in the SpATS model to account for other sources of environmental variation and genotype effects in our sorghum breeding trials.

Thus, the SpATS mixed model for each trial is given by

1 $${\varvec{y}}~={\mathbf{X}}\varvec{\beta}~+~{{\mathbf{X}}_s}{\varvec{\beta}_s}~+~{{\mathbf{Z}}_s}{\varvec{s}}~+~{{\mathbf{Z}}_u}{\varvec{u}}~+~{{\mathbf{Z}}_g}{\varvec{g}}~+~{\varvec{e}},$$

where the vector ***y*** contains the phenotypic observations (grain yield or plant height) arrayed as rows within columns, ***β*** is a vector of fixed terms including the intercept, a check variety effect, and a resolvable block effect, and **X** is the associated design matrix. The fixed (unpenalized) term ${{\mathbf{X}}_s}{\varvec{\beta}_s}$ and the random (penalized) component ${{\mathbf{Z}}_s}{\varvec{s}}$ form the mixed model expression of the smooth spatial surface, i.e., $f({\varvec{r}},~{\varvec{c}})=~{{\mathbf{X}}_s}{\varvec{\beta}_s}{\varvec{~+~}}{{\mathbf{Z}}_s}{\varvec{s}}$, where the vector of random spatial effects ***s*** has covariance matrix **S**. The vector ***u*** comprises the mutually independent sub-vectors of random row and column effects accounting for discontinuous field variation, with design matrix ${{\mathbf{Z}}_u}={\text{[}}{{\mathbf{Z}}_r}{\text{|}}~{{\mathbf{Z}}_c}]$ and covariance matrix ${\mathbf{U}}~{\text{= diag(}}\sigma _r^2{{\mathbf{I}}_r}{\text{, }}\sigma _c^2{{\mathbf{I}}_c}).$ The vector ***g*** contains the random genotypic effects of test-cross hybrids and **Z**_*g*_ is the associated design matrix. We assumed independent genotypic variance, i.e., ***g*** ~ *N*(**0, G**), with ${\mathbf{G}}~{\text{= }}\sigma _g^2{{\mathbf{I}}_g}$. The vector ***e*** consist of spatially independent residuals with distribution ***e*** ~ *N*(**0**, $\sigma _e^2$ **I**). This term, also called nugget, represents the measurement error from each plot.

The SpATS model adopts the P-spline ANOVA (*PS*-ANOVA) formulation proposed by Lee et al. (2013) to represent the 2D P-splines in the mixed model framework. Detailed descriptions of the design matrices ${{\mathbf{X}}_s}$ and ${{\mathbf{Z}}_s}$ and the covariance matrix **S** under this formulation are given in Lee et al. (2013) and Rodríguez-Álvarez et al. (2016a). In this paper, we present the main result of the *PS*-ANOVA parameterization, which is the decomposition of the smooth surface into a sum of linear components and univariate and bivariate smooth functions, such that

2 $$f{\text{(}}\user2{r}{\text{, }}\user2{c}{\text{) = }}\underbrace {{\beta _{{s1}} \user2{r}~{\text{ + }}\beta _{{s2}} \user2{c}~{\text{ + }}\beta _{{s3}} \user2{rc}}}_{{{\mathbf{X}}_{s} \beta _{s} }} + \underbrace {{f_{1} (\user2{r}{\text{) + }}f_{2} (\user2{c}{\text{) + }}h_{3} (\user2{r})\user2{c}{\text{ + }}rh_{4} (\user2{c}{\text{) + }}f_{5} (\user2{r}{\text{,}}\user2{c})}}_{{{\mathbf{Z}}_{s} s}}$$

where the spatial surface is represented by: linear trends across the row (${\beta _{s1}}$) and column (${\beta _{s2}}$) positions and a linear interaction trend (${\beta _{s3}}$); two main smooth trends across rows [ƒ_1_(***r***)] and columns [*ƒ*_2_(***c***)]; two linear-by-smooth interaction terms, where the slope of a linear trend along one covariate (***c*** or ***r***) is allowed to vary smoothly as function of the other covariate [*h*_3_(***r***) or *h*_4_(***c***), respectively]; and ƒ_5_(***r, c***) is the pure smooth-by-smooth interaction between column and row trends.

Under this representation, the vector of random spatial effects ***s*** contains five mutually independent sub-vectors ${{\mathbf{s}}_k}$, with *k* = 1, …, 5 referring to the additive and interaction random components in [2]. Then, the spatial covariance matrix **S** is a direct sum of matrices ${{\mathbf{S}}_k}$, that is ${\mathbf{S}}~={\text{blockdiag(}}{{\mathbf{S}}_1}{\text{,}} \ldots {\text{, }}{{\mathbf{S}}_5})$, where each block ${{\mathbf{S}}_k}$ depends on a specific smoothing parameter ${\lambda _{{s_k}}}$ (see Rodríguez-Álvarez et al. 2016a for details). Within the mixed model framework, each smoothing parameter is determined by REML as the ratio between the residual variance and the corresponding variance of spatial effects, i.e., ${\lambda _{{s_k}}}=~\sigma _e^2 / \sigma _{{s_k}}^2$. Therefore, the smoothness of the spatial surface is tuned by five distinct parameters, applying anisotropic smoothing. The parameterization provides the SpATS model with flexibility to account for both global trends and local variation in the field. Furthermore, the decomposition of $f{\text{(}}{\varvec{r}}{\text{, }}{\varvec{c}}{\text{)}}$ enables a more explicit interpretation of the main patterns of spatial variation.

#### Implementation of the model

The SpATS model with anisotropic smoothing based on the PS-ANOVA approach by Lee et al. (2013) was fitted with the R package (R Development Core Team 2016) SpATS (Rodríguez-Álvarez et al. 2016b), which is publicly available from CRAN (https://cran.r-project.org/package=SpATS). The spatial surface in model [1] was fitted using cubic B-spline bases and second-order penalties, which are commonly used settings in the P-spline framework. Across trials, we used 11 and 31 equally spaced knots for the P-splines in the column and row directions, respectively. In this way, we set approximately one knot for every two rows or columns. Then, the spatial surface contains a total of 425 model parameters to be estimated. These quantities were chosen to provide enough flexibility to the spatial surface. Within the penalized smoothing context, the exact choice of the number of knots is not critical once a certain minimum number of knots is exceeded (Ruppert et al. 2003; Eilers et al. 2015). This number can be equal to the number of rows and columns, i.e., the number of data points in each dimension, or even more. The only limiting factor would be the computational time: the larger the number of knots, the larger the computational effort. It is important to remark that the use of a large number of knots provides flexibility, but in practice, the smoothing parameters are responsible for optimizing the fit to the data.

The estimation procedure implemented in the R package SpATS provides REML-based variance components and computes the empirical best linear unbiased estimates (BLUEs) of fixed effects and the empirical best linear unbiased predictors (BLUPs) of random effects. An important by-product of the procedure is that, for each random effect of the model, an associated effective dimension is computed. The practical implications of the latter concept are considered in the following sections.

#### The effective dimension of the fitted spatial surface

The effective model dimension (ED) or effective number of parameters of a model is a central concept within the P-spline methodology. It is a measure of complexity of the model components and is mainly a function of the smoothing parameters (Eilers et al. 2015). The effective dimension of a model is computed as the trace of the *hat* matrix **H**. If we focus on the spatial part of the SpATS model (1), we have that

$$\tilde f\left( {{\varvec{r}},{\varvec{c}}} \right)~=~{{\mathbf{X}}_s}{\overset{\lower0.5em\hbox{$\smash{\scriptscriptstyle\frown}$}}{\varvec{\beta}} _{s~}}+~{{\mathbf{Z}}_s}{\varvec{\tilde s}}=~{{\mathbf{H}}_\beta }~{\varvec{y}}~+~{{\mathbf{H}}_s}~{\varvec{y}}{\text{,}}$$

where **H**_*β*_ is hat matrix of the fixed component with effective dimension ED_*β*_ = trace(**H**_*β*_) = rank(**H**_*β*_), which is always a constant. More importantly, the total effective dimension of the random (penalized) component of the spatial surface is ED_*s*_ = trace(**H**_*s*_), where **H**_*s*_ is known as the smoother matrix. In this context, the sum of the diagonal elements of **H**_*s*_ expresses the number of parameters effectively involved in the modelling of the spatial surface. From the *PS*-ANOVA decomposition used in the SpATS model, we have that ${{\mathbf{H}}_s}=\sum\nolimits_{k=1}^5 {{{\mathbf{H}}_{{s_k}}}}$, with *k* = 1, …, 5 referring to the additive and interaction smooth components of the spatial trend as detailed in (2). Thus, we can decompose ED_*s*_ as the sum of partial effective dimensions associated with each spatial component:

$${\text{E}}{{\text{D}}_s}{\text{= trace(}}{{\mathbf{H}}_s})=\mathop \sum \limits_{k{\text{=1}}}^5 {\text{trace(}}{{\mathbf{H}}_{{s_k}}})=\mathop \sum \limits_{k{\text{=1}}}^5 {\text{E}}{{\text{D}}_{{s_k}}}.$$

Specifically, when a smoothing parameter ${\lambda _{{s_k}}}=\sigma _e^2/\sigma _{{s_k}}^2$ → ∞, then ${\text{E}}{{\text{D}}_{{s_k}}}$→ 0; while for a value of ${\lambda _{{s_k}}}=\sigma _e^2/\sigma _{{s_k}}^2$ → 0, ${\text{E}}{{\text{D}}_{{s_k}}}$ approaches the maximum value. The upper bound for ${\text{E}}{{\text{D}}_{{s_k}}}$ is determined by the number of knots used to fit the smooth surface. Therefore, ${\text{E}}{{\text{D}}_{{s_k}}}$ serves as a reverse indicator of the smoothness of the corresponding component, i.e., the higher the degree of smoothness (larger value of ${\lambda _{{s_k}}}$), the smaller the number of ${\text{E}}{{\text{D}}_{{s_k}}}$ (see Rodríguez-Álvarez et al. 2016a for details).

Consequently, the total effective dimension ${\text{E}}{{\text{D}}_s}$ can be interpreted as a measure of the magnitude of field variation, with larger values indicating more intense spatial patterns. In addition, the partial effective dimensions ${\text{E}}{{\text{D}}_{{s_k}}}$ are indicative of the relative importance of each spatial component in (2). In this case, the magnitudes of specific ${\text{E}}{{\text{D}}_{{s_k}}}$ will quantify the contribution of the main and interaction spatial trends to the fitted surface, reflecting the complexity of the spatial pattern.

#### Generalized heritability based on the genetic effective dimension

As previously mentioned, an effective dimension connected to each variance component of the SpATS model is computed. The effective dimension associated with the genotypic effects (ED_*g*_) is of particularly interesting for plant breeding. ED_*g*_ = trace(**H**_*g*_) is a measure of the degree of shrinkage imposed on genotypic effects, where **H**_*g*_ is the hat matrix for genotypes. In this case, **H**_*g*_ depends on the regularization parameter ${\lambda _g}={\lambda _g}=\sigma _e^2/\sigma _g^2$ and transforms the observations into predicted genotypic values, such that ${{\mathbf{H}}_g}{\varvec{y}}{\text{}}=~{{\mathbf{Z}}_g}{\varvec{\tilde g}}$ (see Rodríguez-Álvarez et al. 2016a for details). Therefore, ED_*g*_ decreases as shrinkage of genotypic effects increases. Given the properties of the genetic effective dimension, Rodríguez-Álvarez et al. (2016a) proposed a novel expression of heritability:

$${H^2}=\frac{{{\text{E}}{{\text{D}}_g}}}{{{n_g} - l}}$$

where *n*_*g*_ is the number of genotypes and *l* is the number of zero eigenvalues of **H**_*g*_. The authors showed that this definition corresponds to the generalized heritability introduced by Oakey et al. (2006). Furthermore, in the specific situation when genotypic effects are assumed independent (i.e., ignoring pedigree/marker information), and by ignoring the zero eigenvalues, the following equivalence can be stablished:

$${H^2}{\text{ = }}\frac{{{\text{E}}{{\text{D}}_g}}}{{{n_g}}}{\text{ = 1}} - \frac{{\overline {{\text{PEV}}} }}{{\sigma _g^2}},$$

where $\overline {{\text{PEV}}}$ stands for average prediction error variance of genotype BLUPs.

Note that the right-hand term corresponds to the generalized heritability developed by Welham et al. (2010) and is also equivalent to the heritability given by Cullis et al. (2006). Given that our study does not incorporate a genetic relationship matrix, we can profit from the latter equivalence to perform a straightforward comparison between the heritability estimated by the SpATS model and that obtained from the standard mixed models.

### Standard models

Under the standard mixed model framework, we started by fitting a non-spatial model. This baseline model included a random and independent genotypic effect for the test-cross hybrids, a fixed effect for check varieties, a fixed resolvable block effect accounting for the randomization design, and the spatially independent error term ***e*** ~ *N*(**0**, $\sigma _e^2$ **I**). Then, the non-spatial model was extended by searching for the most appropriate spatial model for each case following the approach of Gilmour et al. (1997). The latter model is referred to as the *best standard spatial* (BSS) model.

The general representation of the BSS model can be formulated as

3 $${\varvec{y}}~=~{\mathbf{X}}\varvec{\beta}{\text{ + }}{{\mathbf{X}}_s}{\varvec{\beta}_s}+{{\mathbf{Z}}_s}{\varvec{s}}{\text{ + }}{{\mathbf{Z}}_u}{\varvec{u}}{\text{ + }}{{\mathbf{Z}}_g}{\varvec{g}}~{\text{+ }}\xi ~{\text{+ }}{\varvec{e}},$$

where **X** ***β*** contains the same fixed terms as the non-spatial model. The term ${{\mathbf{X}}_s}{\varvec{\beta}_s}$, in this case, may include linear trends aligned with rows and/or columns to account for global variation, while ${{\mathbf{Z}}_s}{\varvec{s}}{\text{}}$ contains the random part of one or two one-dimensional cubic smoothing splines indexed by row or column positions (see Verbyla et al. 1999 for details). This latter term accounts for additive non-linear global trends. ${{\mathbf{Z}}_u}{\varvec{u}}$, ${{\mathbf{Z}}_g}{\varvec{g}}$, and ***e*** are defined as in the SpATS model (1). Finally, ***ξ*** is the vector of spatially correlated residuals modelling local trend, with distribution $\xi \sim N\left( {{\bf{0}},{\bf{R}}} \right)$. The matrix ${\mathbf{R}}=\sigma _\xi ^2[{\text{AR1}}({\rho _c}) \otimes {\text{AR1(}}{\rho _r}{\text{)}}]$ represents the Kronecker product of first-order autoregressive processes across columns and rows, respectively, and $\sigma _\xi ^2$ is the spatial residual variance.

Following Gilmour et al. (1997), the search for the BSS model was based on diagnostic graphics such as the sample variogram and related plots of residuals. Comparison between candidate models with the same fixed effects was assessed by the REML-likelihood ratio test (REML-LRT). Fixed spatial terms were included in the BSS model when judged significant according to Wald-F test. It is important to note that the BSS model for each trial and trait may represent a simplified version of the full model (3), where the reduced model results from omitting one or more superfluous spatial components.

The standard mixed models were fitted using the ASReml-R package (Butler et al. 2009).

### Comparison of spatial methods

The SpATS model was compared with the non-spatial and the BSS models in terms of meaningful parameters for plant breeding application. The following estimates were considered for comparison:

- Genetic variance ($\sigma _g^2$) and spatially independent residual variance ($\sigma _e^2$).
- Generalized heritability. Estimated following Rodríguez-Álvarez et al. (2016a) for the SpATS model and according to Cullis et al. (2006) for the standard models. These measures are interpreted as broad-sense heritability, which serves as a descriptive measure of precision of a trial, i.e., of the ability to detect genotypic differences among test-cross means.
- Pearson correlations of predicted genotypic values between environments. Given that genotype-by-environment interaction has the same effect on the magnitudes of these correlations for the three models, any increase in their values relative to the non-spatial model will indicate the improvement of precision caused by the spatial models (Qiao et al. 2004; Müller et al. 2010). Only correlations between pairs of environments presenting at least 30 common genotypes were considered.
- Spearman rank correlations between predicted genotypic values from the different models in the same environment. Calculated to compare whether the ranking of genotypes obtained from SpATS and from the standard models differed.

## Results

### Spatial analysis with SpATS

We start with a detailed treatment of the spatial analysis using the SpATS model illustrated with two contrasting trials regarding the intensity and structure of spatial variability. Table 2 presents the ED_*s*_ of the univariate and bivariate spatial smooth components (see Eq. (2)), and their relative contribution to the fitted surface for grain yield in trials DYS05 and DAB08. The magnitudes of the total ED_*s*_ indicate that the spatial variation was more intense in DYS05. This is reflected by the higher ED_*s*_or fitted parameters required to model the underlying field trend (111.2 ED_*s*_ in DYS05 vs 2.1 ED_*s*_ in DAB08). According to the partial ED_*s*_, DYS05 also exhibited a higher complexity in the structure of the spatial surface, where the smooth-by-smooth interaction between trends accounted for most of the field variation (87% of the total ED_*s*_). In contrast, the environmental trend at DAB08 was smoother and less complex as it presented a lower total ED_*s*_ and was mostly captured by main smooth effects across row positions. The zero values of ED_*s*_associated with the linear-by-smooth interactions in DAB08 indicate that these terms were not necessary to model the spatial surface.

**Table 2:** Spatial effective dimensions (ED_*s*_) of the smooth surface components fitted by the SpATS model and its relative contribution (%) for grain yield in two example trials

Spatial smooth terms	DYS05		DAB08	
	ED_*s*_	%	ED_*s*_	%
Additive trends				
*f*_1_(***r***)	3.0	3	1.4	67
*f*_2_(***c***)	4.2	4	0.2	10
Interaction trends				
*h*_3_(***r***)***c***	1.9	2	0.0	0
***r*** *h*_4_(***c***)	5.5	5	0.0	0
*f*_5_(***r, c***)	96.6	87	0.5	24
Total	111.2	100	2.1	100

**Fig. 1:**
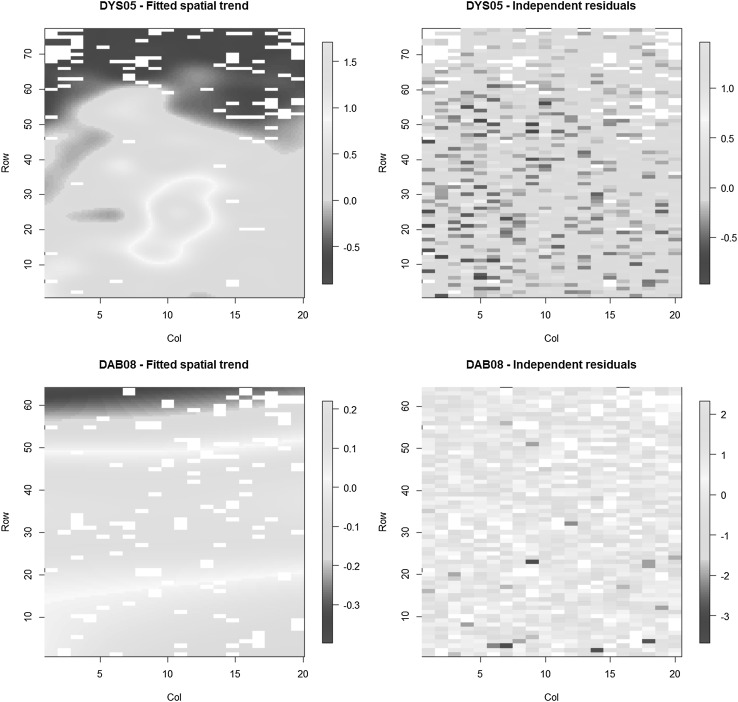
Fitted spatial trend and spatially independent residuals from the SpATS model for grain yield in trials DYS05 (*top*) and DAB08 (*bottom*) plotted against row and column positions. Scales of grain yield variation expressed in t/ha

Figure 1 shows the graphical representations of the fitted spatial trend *f* (***r***, ***c***) and the spatially independent residuals ***e*** for the two example trials, as obtained from the SpATS package. Note that the pictures of the spatial trend use a finer grid than that of the field plots; the P-splines make their computation possible. The spatial surfaces display an irregular patchy pattern in DYS05 and a rather smooth gradient across the field in DAB08. The shape of an evident patch of fertility present in DYS05 was best modelled by considering interactions between column and row trends, as indicated by the partial ED_*s*_ (Table 2). Likewise, the previous interpretation of the spatial trend based on the ED_*s*_ in DAB08 coincides with the plot of the fitted surface, which essentially exhibit a one-dimensional gradient across rows. The inspection of the plots of residuals suggests that the spatial patterns have effectively been removed in both trials by the 2D P-spline surface; hence, these residuals could be considered as true random noise. Other plots of residuals and formal tests could also be used to diagnose outliers, model assumptions, or remaining spatial trends after fitting the spatial model. For the latter purpose, an interesting alternative is the variogram computed from the independent residuals, as proposed by Piepho and Williams (2010). This nugget-based variogram can also be obtained with the SpATS package. The ranges of variation of grain yield data (in t/ha) explained by the fitted trends reflect the magnitude of spatial effects in each trial. The comparison between the scales of spatial and residual site variations provides a clear idea of the relative importance of field trends in these trials. For instance, the range of yield variability due to spatial trends in DYS05 was of similar magnitude to that caused by the spatially independent error, while the amount of variation resulting from the latter term was about tenfold the spatial variability in DAB08 (Fig. 1). Again, the higher relevance of spatial trends for trial DYS05 was also indicated by the total ED_*s*_ presented in Table 2.

In Table 3, we specify the spatial terms of the BSS models for grain yield in DYS05 and DAB08. The connection between these results and those from the analysis with the SpATS model (Table 2) is not straightforward, since the parameterization of both spatial models is different. Assuming that extraneous variations have been adjusted by both models, here, we stress the differences in modelling global and local trends. For instance, according to the standard spatial analysis, in DYS05, there was only a main global trend in the direction of rows, while the column and interaction trends detected by SpATS were apparently modelled as two-dimensional autocorrelated residuals by the BSS model. The main trend across row positions in DAB08 (see Table 2; Fig. 1) seems to be modelled, under the standard approach, by a small autocorrelation across rows and by a value of ${\rho _c}$ close to 1. The latter autocorrelation suggests that the trend across columns is actually confounded with the random row effects (Piepho and Williams 2010; Piepho et al. 2015). Finally, the ratios of spatial variance to residual variance ($\sigma _\xi ^2/\sigma _e^2$) were 2.0 for DYS05 and 0.3 for DAB08, indicating a higher intensity of spatial variation in the former trial (Dutkowski et al. 2002; Zas 2006). The latter results coincide with the interpretation based on the total effective dimensions of the spatial surfaces given in Table 2.

**Table 3:** Spatial terms, estimates of autocorrelations, and variance components for spatially dependent ($\sigma _\xi ^2$) and independent residuals ($\sigma _e^2$) from the *best standard spatial* (BSS) models fitted to grain yield data in two example trials

Trial	BSS model^a^	${\rho _r}$	${\rho _c}$	$\sigma _\xi ^2$	$\sigma _e^2$
DYS05	R + Spl(*r*) + AR1xAR1 + *n*	0.87	0.67	0.103	0.064
DAB08	R + AR1xAR1 + *n*	0.24	0.96	0.201	0.611

^a^R: random row effects; Spl(*r*): cubic smoothing spline indexed by row positions; AR1xAR1: correlated residual modelled as two-dimensional first-order autoregressive process; *n*: spatially independent residual (or *nugget* variance)

**Fig. 2:**
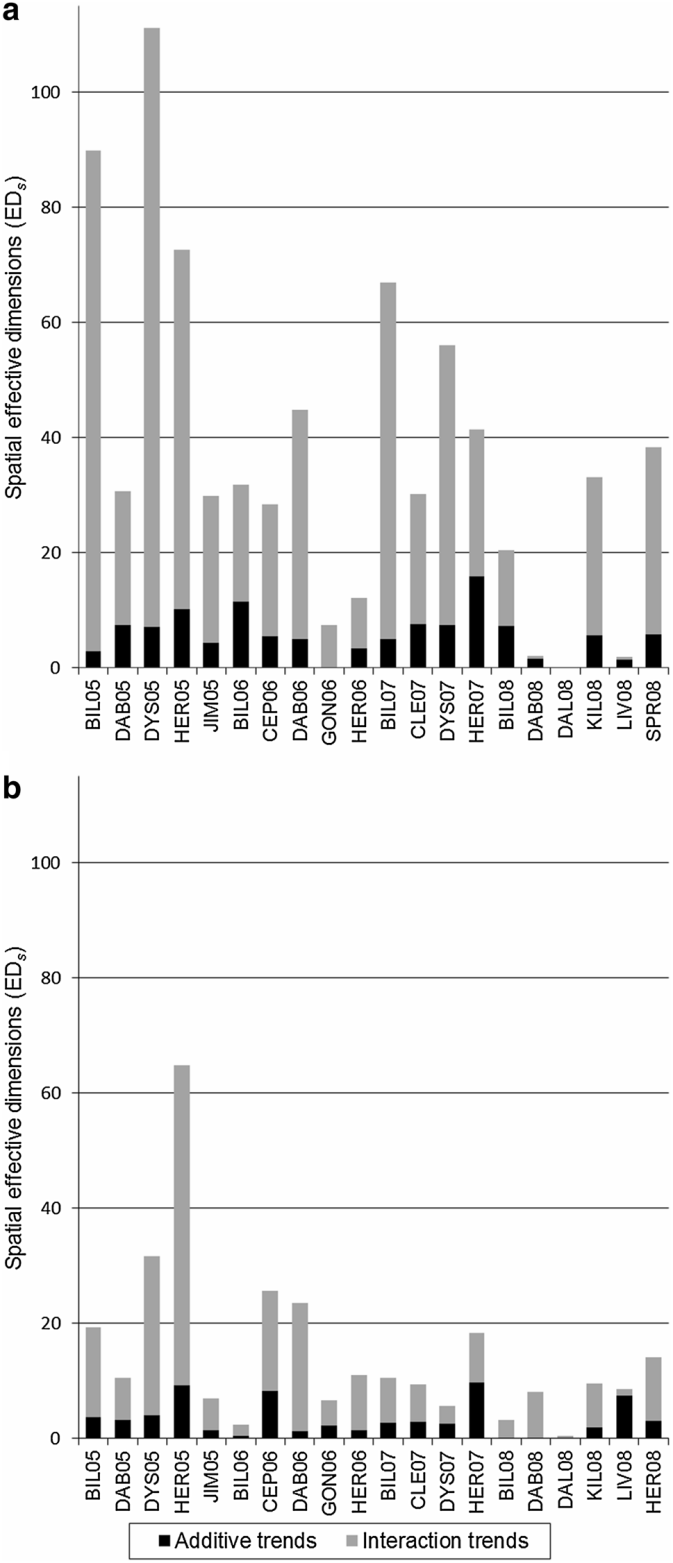
Effective dimensions (ED_*s*_) associated with additive and interaction trends of the spatial surfaces fitted by the SpATS model for grain yield (**a**) and plant height (**b**) in the sorghum breeding trials. Note: data were not available for grain yield at HER08 and for plant height at SPR08

The effective dimensions associated with the fitted spatial trends (ED_*s*_) for all trials and both traits are given in Fig. 2. For simplicity, the partial ED_*s*_ for the five smoothing terms of the SpATS model are grouped as: ED_*s*_ of the additive smooth trends and ED_*s*_ of the interactions between trends. The intensity of spatial variation and the complexity of the fitted surfaces were highly variable across sites and traits. For instance, the environmental trends for grain yield at DYS05 and BIL05 or HER05 for plant height present a large number of ED_*s*_ and a significant contribution of the trend interaction terms, indicating strong and complex patterns of field variation. Others cases, such as DAB08 for grain yield and LIV08 for yield and plant height, show lower total ED_*s*_, reflecting smoother spatial surfaces that were mainly described by additive one-dimensional trends. In general, the intensity of spatial variation for grain yield was higher than for plant height, with median total ED_*s*_ of 31 and 10, respectively. In most instances, the smooth trend interactions represented the major components of the spatial surface. This is reflected by the median ED_*s*_ associated with interaction effects, which were 82 and 79% of the total ED_*s*_ for yield and plant height, respectively. The latter results highlight the importance of modelling interactions between row and column trends and reveals complex structures of field variation in the sorghum data set.

### Standard spatial analysis

A summary of the main features of the BSS models fitted to the sorghum data set is reported in Table 4. Details of the BSS model identified in each of the 20 trials for both traits are presented in Table 5. The results in Table 4 show that most of the trials required terms accounting for global trends, local variation, and nugget effect. Autocorrelations (*ρ*) along rows and columns were predominantly large and similar for both traits, as reflected by their median. Over 80% of the autocorrelation coefficients were larger than 0.60, indicating strong spatial variation that could be interpreted as a combination of large-scale gradients and patchy patterns according to the standard approach. When considering the models with nugget, the importance of the spatial variance relative to the spatially independent residual variance was generally higher for grain yield. The predominance of random noise in plant height measurements indicates that this trait was less influenced by spatial effects in the field. This is consistent with the generally lower effective dimensions of the spatial surfaces estimated for plant height (see Fig. 2). Furthermore, there was a strong positive correlation between the $\sigma _\xi ^2/\sigma _e^2$ ratio and the total ED_*s*_ across the whole data set (*r* = 0.69).

**Table 4:** Number of times the *best standard spatial* (BSS) models for the 20 trials included terms accounting for global and local trends, and median of estimated spatial parameters

	Grain yield	Plant height
Number of trials including:		
Global trend terms	15	11
Correlated residuals (AR1xAR1)	17	17
Nugget effect	17	14
Median of spatial parameters:		
${\rho _r}$	0.82	0.87
$~{\rho _c}$	0.73	0.69
Proportion (%) of correlated error^a^	52	25

^a^Relative to the sum of correlated and independent residual variances

**Table 5:** Details of the *best standard spatial* (BSS) models in each trial for grain yield (GY) and plant height (PH)

Trial	BSS model for GY^a^	BSS model for PH^a^
BIL05	Lin(*r*) + AR1xAR1 + *n*	Lin(*r*) + AR1xAR1(*c*) + *n*
DAB05	R + AR1xAR1 + *n*	Lin(*c*) + AR1xAR1 + *n*
DYS05	R + Spl(*r*) + AR1xAR1 + *n*	Lin(*r*) + AR1xAR1 + *n*
HER05	Lin(*r*) + Spl(*c*) + AR1xAR1 + *n*	Spl(*r*) + Lin(*c*) + AR1xAR1 + *n*
JIM05	C + AR1xAR1 + *n*	R + AR1xAR1 + *n*
BIL06	Spl(*c*) + AR1xAR1 + *n*	C + AR1xAR1
CEP06	R + AR1xAR1 + *n*	Spl(*c*) + AR1xAR1 + *n*
DAB06	C + Spl(*r*) + AR1xAR1 + *n*	AR1xAR1 + *n*
GON06	Spl(*c*) + AR1 + *n*	R + C + AR1
HER06	R + Spl(*r*) + Lin(*c*)	R + C + Lin(*c*) + AR1xAR1 + *n*
BIL07	Lin(*c*) + AR1xAR1 + *n*	R + Spl(*c*)
CLE07	R + Lin(*r*) + AR1xAR1 + *n*	Lin(*c*) + AR1xAR1 + *n*
DYS07	Spl(*c*) + AR1xAR1 + *n*	Lin(*c*) + AR1xAR1 + *n*
HER07	R + Spl(*r*) + Spl(*c*) + AR1xAR1 + *n*	Lin(*r*) + AR1xAR1 + *n*
BIL08	R + C + Spl(*c*)	C
DAB08	R + AR1xAR1 + *n*	Lin(*c*) + AR1 + *n*
DAL08	R + C + Lin(*c*)	C
HER08	–	AR1
KIL08	R + Lin(*r*) + AR1xAR1 + *n*	AR1xAR1 + *n*
LIV08	R + C + AR1xAR1 + *n*	AR1xAR1 + *n*
SPR08	Spl(*c*) + AR1xAR1 + *n*	–

^a^Spl(·): cubic smoothing spline indexed by row (*r*) or column (*c*) positions; Lin(·): linear regression on row (*r*) or column (*c*) positions. R: random row effects; C: random column effects; AR1 and AR1xAR1: correlated residuals modelled as one- and two-dimensional first-order autoregressive process, respectively; *n*: spatially independent residual (nugget effect). Note that all the models included a fixed block effect

### Comparison of SpATS and the standard method

**Fig. 3:**
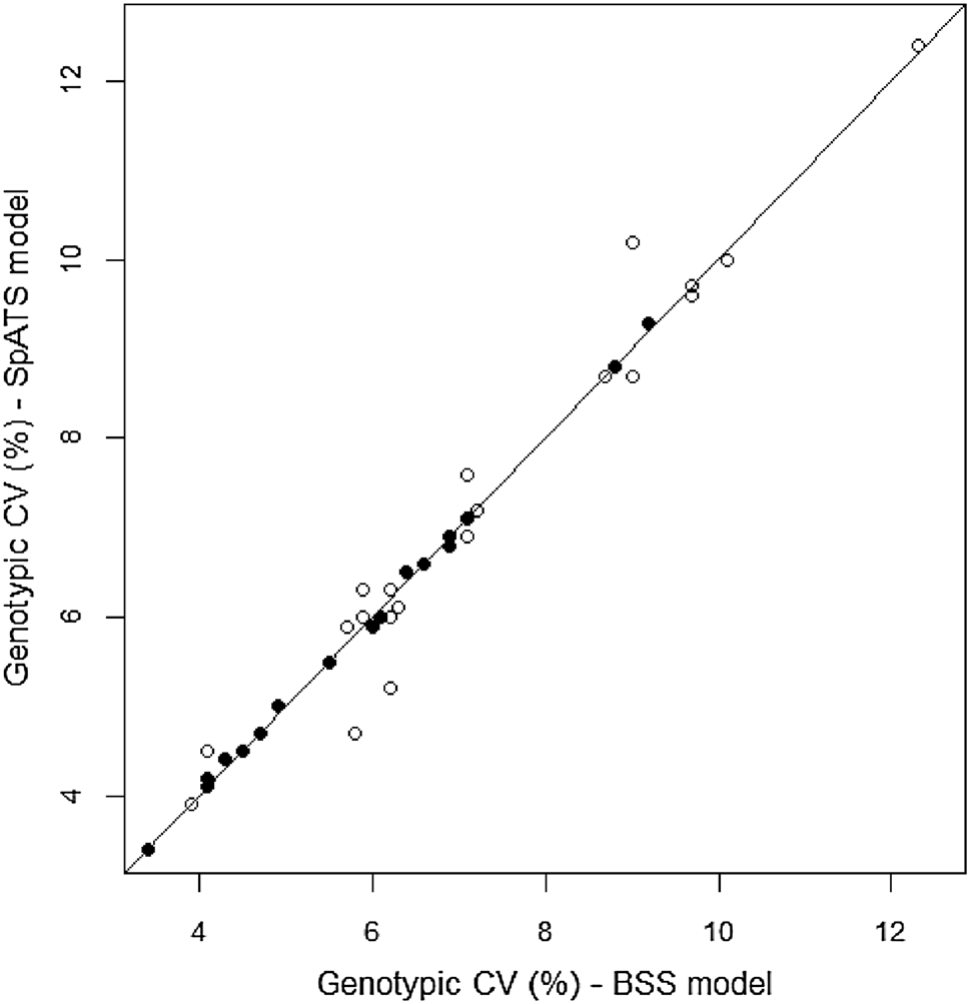
Comparison of genotypic variability estimated by the BSS and the SpATS models, expressed as coefficient of variation (CV), for grain yield (*open circle*), and plant height (*filled circle*). The *diagonal line* indicates identical values

The estimates of trial genetic variability from SpATS and the BSS models were generally similar for both traits (Fig. 3). Small differences were evident for grain yield at some environments, where the estimates increased or decreased from one model to the other without a clear tendency. More marked discrepancies were observed between the genetic variances from the non-spatial model and those from both spatial models for grain yield (not shown). This suggests that ignoring the adjustment for spatial trends in yield data can lead to either overestimating or underestimating the genetic variability.

**Fig. 4:**
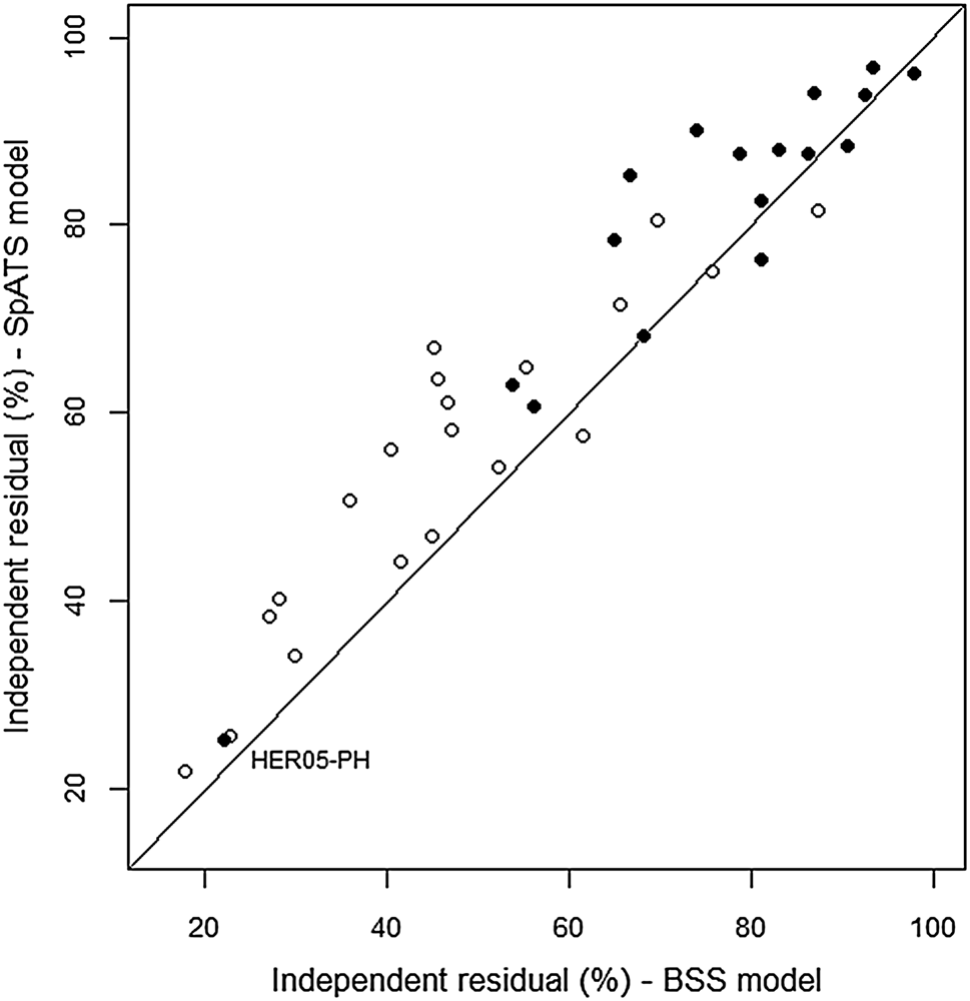
Comparison of spatially independent residual variance ($\sigma _e^2$) from the BSS and the SpATS models, expressed as percentage (%) of the residual variance in the non-spatial model, for grain yield (*open circle*) and plant height (*filled circle*). The *diagonal line* indicates identical values. The labelled data point corresponding to plant height at trial HER05 (HER05-PH) is mentioned in the text

The SpATS model and the BSS models reduced the spatially independent residual variance compared with error variance of the non-spatial model in both traits (Fig. 4). In general, the relative decreases in $\sigma _e^2$ were larger for grain yield, with the spatial models achieving a mean reduction by 49% for grain yield and by 22% for plant height. These reductions reflect the ability of both methodologies to account for field variation not adjusted by the randomization-based model. Exceptionally, the adjustment of spatial trend for plant height caused a large decrease in $\sigma _e^2$ at trial HER05. Note that field trend in this case was particularly important, presenting the highest total ED_*s*_ for plant height and a major contribution of interaction effects (see Fig. 2). In general, the BSS models estimated smaller values of $\sigma _e^2$ compared to the SpATS model. The spatially independent component from SpATS and the BSS models represented, on average, 66 and 60% of the residual variance from the non-spatial model, respectively.

**Fig. 5:**
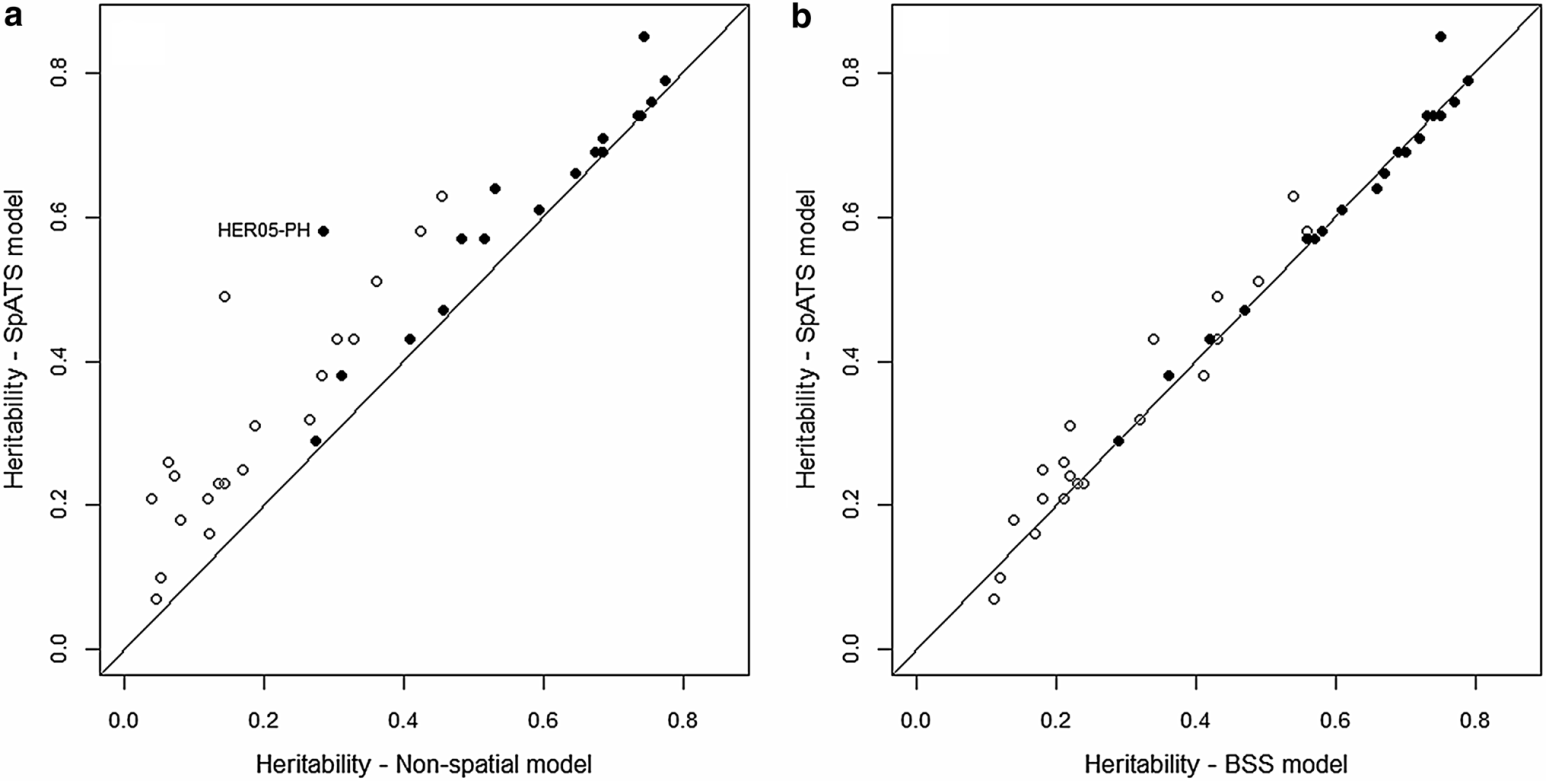
Comparison between estimates of heritability from the non-spatial and SpATS models (**a**), and from SpATS and the BSS models (**b**) for grain yield (*open circle*) and plant height (*filled circle*). The *diagonal lines* indicate identical values. The labelled data point corresponding to plant height at trial HER05 (HER05-PH) is mentioned in the text

Figure 5a shows the changes in the estimates of trial heritability from the non-spatial model to the SpATS model. The spatial method increased the heritability in most instances, with levels of improvement in precision being generally higher for grain yield. Not surprisingly, a remarkable increase in heritability was also achieved for plant height in HER05 after fitting trends with SpATS. Trial heritabilities estimated by both spatial methods were very consistent for plant height (Fig. 5b). However, more variation in the estimates was observed for grain yield, where similar or slightly higher heritabilities were obtained with the SpATS model in most trials. Finally, notice that heritabilities were, in general, lower for grain yield, which was the trait affected by stronger spatial variation (as inferred from the total ED_*s*_ in Fig. 2).

**Fig. 6:**
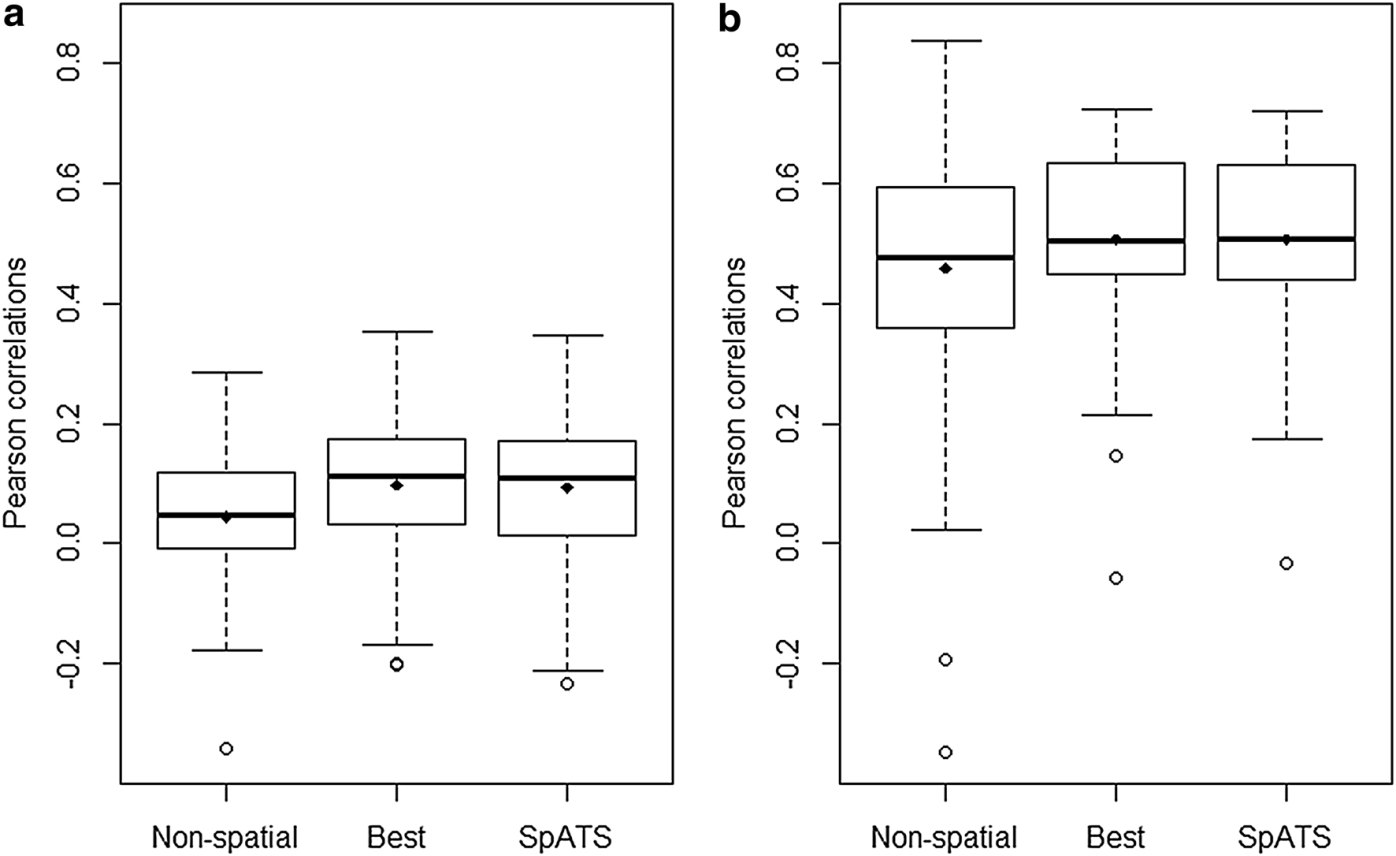
Correlations of genotype BLUPs between environments from each model for grain yield (**a**) and plant height (**b**)

The Pearson correlations of genotype BLUPs between environments obtained from the two spatial methods were, on average, slightly higher than those obtained from the non-spatial model in both traits (Fig. 6). The mean correlations for grain yield increased from 0.04 to 0.10 and 0.09 after applying the BSS models and SpATS, respectively (Fig. 6a). For plant height, both spatial models caused a mean increase of 0.05 in the correlations, changing from 0.46 to 0.51 (Fig. 6b). At the same time, the spatial methods reduced the variation of estimated correlations for the latter trait. The higher mean correlations between environments in plant height reflect a lower influence of genotype-environment interaction.

**Fig. 7:**
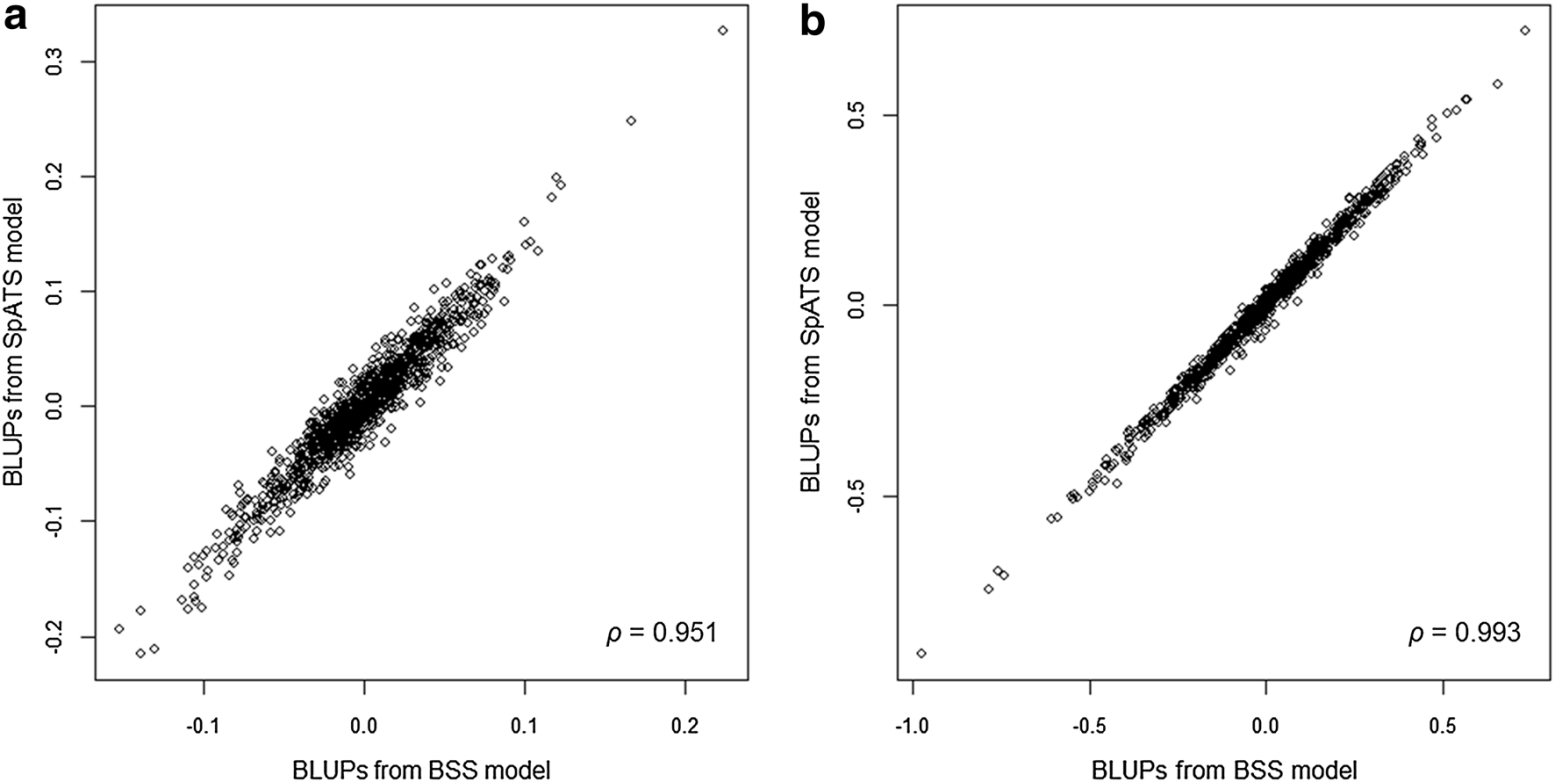
Genotype BLUPs from the BSS model and the SpATS model and Spearman rank correlations (*ρ*) for grain yield in trials DYS05 (**a**) and DAB08 (**b**)

For illustration purpose, Fig. 7 presents the BLUPs of genotype effects from SpATS and the BSS models for grain yield in the example trials DYS05 and DAB08. Differences in the rankings were small for both environments. However, changes in the order of genotypes were more evident at DYS05 (Fig. 7a), an environment where, as previously noted, the nature of spatial variation was more complex. The predicted rankings established by both spatial methods were also consistent for the rest of the data set, with mean Spearman correlations across trials of 0.970 for grain yield and 0.989 for plant height. As expected, the rankings of genotype were more dissimilar between SpATS and the non-spatial models. Rank correlations for yield between these models ranged from 0.500 at DYS05 (where ED_*s*_ = 111.2) to 0.926 at DAL08 (where ED_*s*_ = 0.0), with a mean value of 0.802. In the case of plant height, correlations were generally higher, varying from 0.767 at HER05 (where ED_*s*_ = 64.8) to 0.988 at DAL08 (where ED_*s*_ = 0.4) and a mean value of 0.944.

As suggested by one of the reviewers, we tried to implement a single-step model selection strategy with the standard method using the full model (3) across our data set. Convergence problems were evident in 8 out of 20 trials for grain yield and in 10 out of 20 trials for plant height. It was possible to decrease the rate of failure by relaxing convergence criteria. However, given that we know the full model was a misspecified one, tuning strategies should not be used to get convergence. The failures to converge reflected identifiability problems for the situation when the AR1xAR1 structure, global trend terms, and the nugget are included in the same model. In contrast, the SpATS model did not suffer from this confounding difficulty; the three types of field variation were fitted in a stable way.

## Discussion

### Spatial analysis with SpATS

This study presented the SpATS model as a suitable alternative to the standard spatial models for the adjustment of field trends in sorghum genetic trials. We reported a first application of the new spatial model to a real and extensive plant breeding field testing. This method fits a smooth surface to account for all sources of continuous environmental variation. The mixed model representation of SpATS features the joint modelling of additive one-dimensional trends plus interactions between trends in the row and column directions. Moreover, the model specification assigns different degrees of smoothing to each additive and interaction effect by means of specific smoothing parameters. These weighting terms, which are automatically tuned by REML-based variance components, shrink irrelevant effects to optimize the fit of the spatial surface.

We have stressed the practical importance of the effective dimension of the model as an integral part of spatial analysis with SpATS. This study highlights how ED_*s*_ can be used to interpret the intensity and the structure of spatial variation. The ED_*s*_ is a very appealing tool to quantify the magnitude of spatial effects, reflecting the amount of smoothing of the spatial surface and allowing an easy identification of the main patterns of field heterogeneity. Furthermore, the genetic effective dimension was used to compute a generalized heritability in the context of analysis with SpATS (Rodríguez-Álvarez et al. 2016a). This novel expression of heritability is valid for more general situations commonly found in plant genetic trials, *e.g*., when data are unbalanced and/or when residuals are spatially correlated. Equivalent definitions of generalized heritability were also proposed by Cullis et al. (2006) and Welham et al. (2010) in the context of standard mixed model analyses.

To subject the new method to a hard evaluation, we analysed large-scale sorghum breeding trials arrayed as partially replicated (*p*-rep) designs (Cullis et al. 2006). These experiments are characterised by the absence of the traditional blocking factors, allowing very few or no design features to be retained in the randomization-based model. Moreover, the use of partially replicated experiments assumes that field trend affecting unreplicated genotypes can be properly predicted by the spatial model (Payne 2006). Consequently, the analysis of *p*-rep designs requires the inclusion of spatial parameters as an essential add-on component for an efficient testing of genetic material. The results of our study demonstrate the effectiveness of SpATS to account for spatial trend and predict adjusted genotypic values under these circumstances. The SpATS model adjusts a continuous surface across the whole field. A more refined modelling could consider a discontinuity in spatial trend by fitting a different surface within each block. Even though the former approach is more conservative, we consider that it reflects the structure of trial design and should be a realistic model for most commonly used experiments in plant breeding. Furthermore, the smoothness of the spatial surface fitted by SpATS is controlled by five different terms, providing enough flexibility for an appropriate fit of the spatial trend.

### Comparison of SpATS and the standard method: parameterization

The SpATS model presents similarities with the full formulation of the standard spatial model (see Eq. 3). Both models contains two one-dimensional spline terms, each one fitted as the sum of a fixed linear trend and a random non-linear component. In addition, discontinuous spatial trends are accounted for by random row and column effects in both cases. However, there is a major difference between both models when accounting for the remaining spatial variation. The standard model fits a separable AR1 process, whereas SpATS uses P-spline interaction terms.

This difference in parameterization affects the way in which SpATS and the standard methods model field variation. Under the standard mixed model approach, gradients across the field can be adjusted by blocking factors and by one-dimensional polynomials or splines along rows and columns. However, more complex two-dimensional gradients that do not align well with row and column directions are expected to affect field trials as well. The structure of spatial variation found in our data set (Fig. 2) and in the previous studies of agricultural and forest field trials demonstrate that fitting only additive gradients in one dimension may result in insufficient modelling of global trend (Federer 1998; Fu et al. 1999; Taye and Njuho 2008). It is in principle possible to extend the standard spatial model with additional fixed terms, like a linear x linear interaction term (Federer 1998), and random terms, like a smoothing spline interaction term, but these extensions were never used under the standard approach and are prone to cause problems (Gilmour 2000). In this research, we showed that the SpATS model is able to account for intricate patterns of large-scale variation by explicitly modelling the interactions between global trends along rows and columns.

A common practice in spatial analysis is to fit an autoregressive model, originally proposed to adjust for local trend, and assume that it is flexible enough to also account for global trend (Zimmerman and Harville 1991; Dutkowski et al. 2006; Piepho et al. 2008). A more conservative approach considers that the underlying spatial correlation is likely to hold only within blocks and that large-scale trend is partially accounted by blocking factors (Williams et al. 2006; Piepho and Williams 2010). However, continuous non-stationary trends across the whole field can be better fitted by specific spatial terms in the model, as was suggested in the seminal paper by Gilmour et al. (1997). Following their approach, we found that additional terms accounting for global variation could not have been ignored in most of the sorghum trials and for both traits (see Table 4). Several studies using real and simulated data have shown that underfitting global trend may cause the variance of treatment differences to be underestimated (Zimmerman and Harville 1991; Brownie et al. 1993; Brownie and Gumpertz 1997). This false improvement in precision can be particularly negative in plant breeding trials as it reduces the efficiency of selection decisions. Furthermore, Brownie and Gumpertz (1997) reported that local trend is overestimated in presence of unaccounted large-scale trend. Given that global and local model terms are actually “competing” to fit part of the same spatial variation, the estimated covariance parameters will vary according to the global terms included in the spatial model. This inconsistency in the estimates of autocorrelations was also observed in our study during the search of the BSS models (not shown). The aforementioned situation raises the issue of parameter identification when both global and local trend are trying to be fitted. Therefore, spatial parameters should be interpreted with special care under the standard approach. Conversely, the new spatial method based on 2D P-splines simplifies the problem of spatial model identification by always modelling all types of field trend as a single continuous process. This unified modelling avoids the necessity of distinguishing between global and local trend. Both forms of continuous variation are simultaneously fitted by the flexible interaction surface with anisotropic smoothing. As a result, SpATS provides a straightforward representation of the spatial trend that is easy to interpret. Moreover, the ANOVA-type decomposition of the smooth surface facilitates the characterization of the spatial trend, providing additional insight into the structure of field variation.

Another difference between both spatial methods was evident regarding the estimation of the residual variance. In our data set, the standard spatial models exhibited a clear tendency to estimate smaller spatially independent components than the SpATS model (Fig. 4). The same discrepancy was reported by Rodríguez-Álvarez et al. (2016a) in a simulation study where they analysed data generated according to different autoregressive models with nugget. These authors showed that, when the autocorrelations are large (*ρ*_*r*_ = *ρ*_*c*_ = 0.9), the SpATS model provides relatively accurate estimates of the random error variance, whereas the autoregressive model tends to underestimate this term. The possibility of confounding the spatial component with the nugget variance when fitting autoregressive models in field trials was also reported by Cullis et al. (1998) and extensively discussed in Piepho et al. (2015). Given the large autocorrelations estimated in most of our sorghum trials, we may suggest that SpATS performed generally better in identifying the true spatially independent residuals, while the BSS models were actually modelling part of the random error as spatially correlated data. This potential confounding of parameters in autoregressive and other non-linear spatial models with nugget causes frequent convergence problems (e.g., Dutkowski et al. 2006; Müller et al. 2010; Liu et al. 2015; Rodríguez-Álvarez et al. 2016a). When convergence cannot be reached, one could fall back to alternative models without nugget effects (Müller et al. 2010; Leiser et al. 2012). This strategy is far from attractive given that the potential best fitting model would be deliberately ignored. Furthermore, our research (Table 3) and other studies demonstrated that a spatially independent component accounting for measurement error is frequently required (e.g., Cullis et al. 1998; Qiao et al. 2000; Liu et al. 2015). In contrast to the standard spatial modelling approach, the SpATS model always fits an random error variance on top of the spatial surface and, in our experience, it always converges readily; see also Rodríguez-Álvarez et al. (2016a). As a reviewer suggested, in addition to the identifiability issues mentioned above for the standard approach, it cannot be excluded that the difference in convergence performance between SpATS and the standard models may be related to the standard method using a covariance structure that is non-linear in the variance parameters, while the covariance structure of SpATS is linear in the parameters. Further study is required here.

### Comparison of SpATS and the standard method: performance

The comparison between SpATS and the best fitting standard spatial models revealed a similar performance for the evaluation criteria considered in this paper. Besides the differences discussed above, both methods caused similar reductions in the spatially independent residual variance compared with the error of the non-spatial model. This changes indicate the magnitude of spatial variation adjusted by the spatial models for both traits. The generally large decreases in the random error component (>30%) obtained for grain yield reflect that strong spatial trends affected this trait in most trials (Stroup et al. 1994; Yang et al. 2004). The lower reductions observed for plant height could be related to the dominant presence of random environmental variation (see Table 3). Interestingly, the same inferences can be drawn by considering the higher ED_*s*_ that were usually associated with grain yield trends (Fig. 1). The larger number of parameters effectively estimated by SpATS to better approximate the underlying spatial surface reflected the higher intensity of field trends for grain yield data. The ability of the ED_*s*_ to indicate the relative importance of spatial variation was evidenced by the strong positive association between the number of ED_*s*_ and the ratio of spatial to spatially independent variance from the standard models.

In general, the estimates of genetic variance from the SpATS model were comparable to those obtained by the BSS models. The inconsistencies between both models observed in some cases may result from the impossibility to clearly identify the genetic and the environmental variation in presence of spatial correlation. Several simulation studies have shown that unadjusted patchiness in the field may inflate the genetic variance (e.g., Loo-Dinkins et al. 1990; Magnussen 1993, 1994). This identification problem was apparent across candidate BSS models, where the autoregressive models ignoring the nugget estimated higher trial genetic variances than the better-fitting models using nugget (data not shown). The overestimation of genetic variation when adjusting autoregressive models without nugget was also reported by Dutkowski et al. (2002) in tree breeding trials and by Rodríguez-Álvarez et al. (2016a) using simulated data. In addition, the latter authors showed that SpATS produced more accurate estimates of genetic variance, which were highly consistent with those obtained from the best fitting standard model including the nugget. However, more extensive assessments of the SpATS model would be still necessary with respect to the validity of estimates when spatial variation is present.

Several studies considered the changes in heritability to measure the impact of alternative models on the efficiency of plant breeding evaluations (e.g., Smith et al. 2001; Welham et al. 2010; Sarker and Singh 2015). Following this approach, we used the generalized heritability to compare the performance of the SpATS model and the standard spatial models. The adjustment of spatial trends with the new spatial model led to levels of heritability equivalent to the standard models in all the sorghum trials. The increases in grain yield heritability compared to the randomization-based model were broadly consistent with the results from standard spatial analysis of sorghum breeding trials in West Africa (Leiser et al. 2012). The improvement in precision, measured as the increase in the correlation of genotype predictions between environments, was generally the same for both spatial methods. Similar magnitudes of improvements through standard spatial analysis were previously reported by Leiser et al. (2012) in sorghum, but smaller increases were achieved for wheat, sugar beet, and barley breeding trials (Qiao et al. 2004; Müller et al. 2010).

The analysis with SpATS affected the predictions of genotypic values, as the ranking of genotypes changed after modelling the spatial trends. A bigger impact on genotype ranks was usually observed in cases where the fit of a smooth surface produced larger increases in broad-sense heritability. Our results showed high consistency in the ranking of genotypes predicted by the SpATS model and the BSS models for all cases. This indicates that the use of the new spatial method would hardly produce changes in selection decisions compared to the more refined spatial models. The consistent but small changes in predicted rankings may be a consequence of the differences discussed above related to how both spatial methods accommodate global and local trends.

### Comparison of SpATS and the standard method: modelling strategy

In this paper, we used a single-step modelling strategy to perform the definite spatial analysis in every trial. Furthermore, the same SpATS model was applied for individual-trial analysis across the whole data set. This approach differs from the common modelling procedure based on sequential fitting of alternative spatial models for each trial. The latter practice may be a limitation for efficient routine application given that several model selection steps are required to arrive at a final spatial model. To perform the standard spatial analysis in the present paper, we inspected alternative AR1 models. However, the number of potential candidate models increases if other spatial covariance structures are also considered. A strategy to simplify the model selection process may be to restrict the number of candidate spatial models, potentially reducing the efficiency of analysis. A remarkable attempt to maximize efficiency of plant breeding trials through standard spatial analysis was reported by Leiser et al. (2012), who fitted 91 different models for each trial to identify the best models in 17 environments. Unfortunately, these efforts for further modelling usually result in modest benefits relative to simpler models. Alternative spatial methods based on kriging are also time-consuming and difficult to apply in practice (Zas 2006; de la Mata and Zas 2010).

Our approach using SpATS accounted for all types of spatial variation by fitting a single model rather than using a multi-step modelling procedure. Under this simplified strategy, model selection steps required to identify the appropriate spatial correlation and/or global trend terms are not needed; both local and global trends are automatically modelled in a single step by the smooth surface. The SpATS approach relies on the estimation procedure to effectively reduce the influence of the smooth surface components that are not needed. This implicit model selection is automatically tuned by specific smoothing parameters (or penalties) and is reflected in the ED_*s*_. Accordingly, after convergence, the ED_*s*_ of unimportant components will tend to zero, meaning that these terms are not contributing to the complexity of the spatial model. The new method also simplifies the practice of using diagnostic graphics, such as variograms, to guide model selection. The reason is that the selections steps required to fit global and local trends under the standard method are reduced to one with SpATS, and thus, the diagnostic plots associated to those steps are essentially skipped. Random row and column effects were fitted by default in our SpATS model, as discontinuous spatial effects were also present in most cases (data not shown). The inclusion of these effects in a default spatial model is justified by the frequent existence of non-smooth effects caused by blocking factors or extraneous variation (e.g., Piepho and Williams 2010; Liu et al. 2015). We set the same number of equally spaced knots in each dimension of the 2D P-spline for every trial. These quantities were chosen to be so many as to ensure ample flexibility to the smoother. Other studies on spatial analysis with P-splines reported that models using different numbers of knots produced similar fits and results (Cappa and Cantet 2007; Cappa et al. 2011). Moreover, Eilers et al. (2015) demonstrated that, once a sufficient number of knots has been chosen, optimizing their quantity is not worthwhile, because the smoothing parameters will regulate the smoothness of the fit to optimize the bias-variance trade-off.

For the present research, we have used a general SpATS model considering the design and treatment factors of our data set. However, it is noteworthy that the mixed model formulation of SpATS enables more refined model building/selection according to specific situations. For instance, having an ED of zero is equivalent to an associated variance component being zero. It implies that we could use any tests that evaluate the relative fit of a variance model (e.g., REML-LRT, AIC) to perform model selection.

The results from this study showed that the SpATS model performed comparably to more refined and site-specific spatial models. One advantage of the novel method is that all types of continuous spatial variation and genetic effects can be modelled simultaneously in a single modelling step. As Dutkowski et al. (2006) pointed out, this approach should be superior to fitting all terms in a multi-step process as it will avoid parameter identification problems derived from confounding spatial heterogeneity with genetic heterogeneity due to aggregation of related genotypes. An additional benefit is that the SpATS model may be useful to improve the efficiency of two-stage analysis of multi-environment trials (MET). The reason is that the same flexible model can be fitted in the first stage to account for the spatial surfaces of all the trials, obtaining adjusted genotype means to be used in the second stage. The gain in speed of analyses using the new method results from the fact that less computational steps would be needed to identify an appropriate spatial model for each trial.

## Conclusion

The SpATS model provided a flexible and efficient alternative to account for spatial patterns in the sorghum breeding field trials. The performance of the new model was equivalent to the more elaborate standard spatial models when considering the improvement in precision and the predictions of genotypic values. The suitability of SpATS was consistent across trials and traits exhibiting different magnitudes of heritability and complexity of spatial variation. A major advantage of the new model over existing techniques is that global and local trends are jointly modelled by the smooth surface. Moreover, we used a general SpATS model to adequately fit all experiments, which avoids the examination of several candidate models for each trial. Given the results of this study, the use of the new method should be considered as a simple and effective strategy to optimize the practical application of spatial analysis in plant breeding trials.

### Author contribution statement

FvE conceived the research. JV, DJ, MM, and FvE designed the research. JV performed statistical analyses and wrote the manuscript. MXRA, MB, and PE supported the application and understanding of the SpATS methodology and corresponding R package. DJ coordinated the field trials and the data collection. MXRA, MB, MM, and FvE edited the manuscript. All authors read and approved the final manuscript.
